# Supplementary material for: Electroencephalogram synchronization measure as a predictive biomarker of Vagus nerve stimulation response in refractory epilepsy: A retrospective study
Source: PLoS One. 2024 Jun 11;19(6):e0304115. doi: 10.1371/journal.pone.0304115 (PMC11166337; doi:10.1371/journal.pone.0304115)
Supplement: S1 Table — (DOCX) [file pone.0304115.s001.docx]

**S1 Table. Linear Mixed Models Using wPLi as Dependent Variable in Delta Band With and Without Covariables.**

Model with covariables

| Explanatory variable | Para-  meter | Estimate | Standard error | t value | Pr (>\|t\|) |  |
| --- | --- | --- | --- | --- | --- | --- |
| (Intercept) | β0 | 0.3970566 | 0.0359326 | 11.050 | 2.41x10^-11^ | *** |
| State (wakefulness/sleep) | β1 | -0.0212028 | 0.0099415 | -2.133 | 0.0407 | * |
| Response to VNS | β2 | -0.0180504 | 0.0188051 | -0.960 | 0.3431 |  |
| Number of ASM | β3 | 0.0098252 | 0.0105155 | 0.934 | 0.3588 |  |
| Sex | β4 | 0.0056411 | 0.0152942 | 0.369 | 0.7153 |  |
| Patient’s age at EEG | β5 | 0.0001713 | 0.0006529 | 0.262 | 0.7951 |  |
| Take of BZD | β6 | 0.0181021 | 0.0214059 | 0.846 | 0.4032 |  |
| Type of epilepsy | β7 | 0.0000239 | 0.0169355 | 0.001 | 0.9989 |  |
| Epilepsy duration | β8 | 0.0003871 | 0.0005468 | 0.708 | 0.4855 |  |
| Localization of epilepsy | β9 | 0.0009637 | 0.0130259 | 0.074 | 0.9416 |  |
| Interaction between the state and the response. | β10 | 0.0206664 | 0.0174262 | 1.186 | 0.2444 |  |

*: p<0.05　　**: p<0.01　　***: p<0.001

Abbreviations: VNS (vagus nerve stimulation), EEG (electroencephalogram), ASM (antiseizure medication), BZD (benzodiazepine)

**Final model**

| Explanatory variable | Para-  meter | Estimate | Standard error | t value | Pr (>\|t\|) |  |
| --- | --- | --- | --- | --- | --- | --- |
| (Intercept) | β0 | 0.443082 | 0.008577 | 51.661 | <2x10^-16^ | *** |
| State (wakefulness/sleep) | β1 | -0.023128 | 0.009118 | -2.537 | 0.0157 | * |
| Response to VNS | β2 | -0.016134 | 0.015262 | -1.057 | 0.2946 |  |
| Interaction between the state and the response. | β3 | 0.018166 | 0.016225 | 1.120 | 0.2703 |  |

*: p<0.05　　**: p<0.01　　***: p<0.001

Abbreviations: VNS (vagus nerve stimulation), EEG (electroencephalogram), ASM (antiseizure medication), BZD (benzodiazepine)
