# Supplementary material for: Electroencephalogram synchronization measure as a predictive biomarker of Vagus nerve stimulation response in refractory epilepsy: A retrospective study
Source: PLoS One. 2024 Jun 11;19(6):e0304115. doi: 10.1371/journal.pone.0304115 (PMC11166337; doi:10.1371/journal.pone.0304115)
Supplement: S2 Table — (DOCX) [file pone.0304115.s002.docx]

**S2 Table. Linear Mixed Models Using wPLi as Dependent Variable in Alpha Band With and Without Covariables.**

Model with covariables

| Explanatory variable | Para-  meter | Estimate | Standard error | t value | Pr (>\|t\|) |  |
| --- | --- | --- | --- | --- | --- | --- |
| (Intercept) | β0 | 0.3664265 | 0.0447621 | 8.186 | 9.28x10^-9^ | *** |
| State (wakefulness/sleep) | β1 | 0.0303440 | 0.0153778 | 1.973 | 0.0571 | . |
| Response to VNS | β2 | -0.0055522 | 0.0246647 | -0.225 | 0.8229 |  |
| Number of ASM | β3 | 0.0034138 | 0.0130662 | 0.261 | 0.7960 |  |
| Sex | β4 | 0.0227216 | 0.0189489 | 1.199 | 0.2416 |  |
| Patient’s age at EEG | β5 | 0.0010398 | 0.0008093 | 1.285 | 0.2105 |  |
| Take of BZD | β6 | 0.0250464 | 0.0275016 | 0.911 | 0.3691 |  |
| Type of epilepsy | β7 | 0.0112207 | 0.0209840 | 0.535 | 0.5975 |  |
| Epilepsy duration | β8 | 0.0000782 | 0.0006776 | 0.115 | 0.9090 |  |
| Localization of epilepsy | β9 | -0.0023097 | 0.0161452 | -0.143 | 0.8874 |  |
| Interaction between the state and the response. | β10 | -0.0368851 | 0.0269803 | -1.367 | 0.1811 |  |

*: p<0.05　　**: p<0.01　　***: p<0.001

Abbreviations: VNS (vagus nerve stimulation), EEG (electroencephalogram), ASM (antiseizure medication), BZD (benzodiazepine)

**Final model**

| Explanatory variable | Para-  meter | Estimate | Standard error | t value | Pr (>\|t\|) |  |
| --- | --- | --- | --- | --- | --- | --- |
| (Intercept) | β0 | 0.43318 | 0.01190 | 36.408 | <2x10^-16^ | *** |
| State (wakefulness/sleep) | β1 | 0.02871 | 0.01379 | 2.083 | 0.0444 | * |
| Response to VNS | β2 | -0.01198 | 0.02117 | -0.566 | 0.5733 |  |
| Interaction between the state and the response. | β3 | -0.03420 | 0.02453 | -1.394 | 0.1718 |  |

*: p<0.05　　**: p<0.01　　***: p<0.001

Abbreviations: VNS (vagus nerve stimulation), EEG (electroencephalogram), ASM (antiseizure medication), BZD (benzodiazepine)
