# Supplementary material for: Electroencephalogram synchronization measure as a predictive biomarker of Vagus nerve stimulation response in refractory epilepsy: A retrospective study
Source: PLoS One. 2024 Jun 11;19(6):e0304115. doi: 10.1371/journal.pone.0304115 (PMC11166337; doi:10.1371/journal.pone.0304115)
Supplement: S3 Table — (DOCX) [file pone.0304115.s003.docx]

| Explanatory variable | Para-  meter | Estimate | Standard error | t value | Pr (>\|t\|) |  |
| --- | --- | --- | --- | --- | --- | --- |
| (Intercept) | β0 | 0.3250736 | 0.0509263 | 6.383 | 9.07x10^-6^ | *** |
| Future percentage of seizure reduction (after one year of VNS) | β1 | -0.0006608 | 0.0003995 | -1.654 | 0.1176 |  |
| Patient’s age at EEG | β2 | 0.0021879 | 0.0008599 | 2.544 | 0.0216 | * |
| Epilepsy duration | β3 | -0.0004810 | 0.0007983 | -0.603 | 0.5553 |  |
| Sex | β4 | 0.0133890 | 0.0222810 | 0.601 | 0.5563 |  |
| *Localization of epilepsy* | *β5* | *0.0045962* | *0.0192274* | *0.239* | *0.8141* | *Higher p-value* |
| Number of ASM | β6 | 0.0186846 | 0.0143863 | 1.299 | 0.2124 |  |
| Take of BZD | β7 | -0.0192962 | 0.0341204 | -0.566 | 0.5796 |  |
| Type of epilepsy | β8 | 0.0138464 | 0.0235164 | 0.589 | 0.5642 |  |

S3 Table. Models of multiple Regression Analysis Using wPLI as a Dependent Variable in Alpha Band in Sleep.

Model 1: 8 variables

Adjusted R-squared of the model: 0.2009

p-value: 0.1612

*: p<0.05　　**: p<0.01　　***: p<0.001

Abbreviations: VNS (vagus nerve stimulation), EEG (electroencephalogram), ASM (antiseizure medication), BZD (benzodiazepine)

Model 2: 7 variables

Adjusted R-squared of the model: 0.2452

p-value: 0.09836.

| Explanatory variable | Para-  meter | Estimate | Standard error | t value | Pr (>\|t\|) |  |
| --- | --- | --- | --- | --- | --- | --- |
| (Intercept) | β0 | 0.3246637 | 0.0494659 | 6.563 | 4.83x10^-6^ | *** |
| Future percentage of seizure reduction (after one year of VNS) | β1 | -0.0006798 | 0.0003805 | -1.787 | 0.0918 | . |
| Patient’s age at EEG | β2 | 0.0022292 | 0.0008187 | 2.723 | 0.0145 | * |
| Epilepsy duration | β3 | -0.0005004 | 0.0007718 | -0.648 | 0.5254 |  |
| Sex | β4 | 0.0144345 | 0.0212330 | 0.680 | 0.5058 |  |
| Number of ASM | β5 | 0.0193894 | 0.0136849 | 1.417 | 0.1746 |  |
| *Intake of BZD* | *β6* | *-0.0184895* | *0.0329981* | *-0.560* | *0.5826* | *Higher p-value* |
| Type of epilepsy | β7 | 0.0203866 | 0.0235164 | 0.804 | 0.4326 |  |

*: p<0.05　　**: p<0.01　　***: p<0.001

Abbreviations: VNS (vagus nerve stimulation), EEG (electroencephalogram), ASM (antiseizure medication), BZD (benzodiazepine)

Model 3: 6 variables

Adjusted R-squared of the model: 0.2739

p-value: 0.06077.

| Explanatory variable | Para-  meter | Estimate | Standard error | t value | Pr (>\|t\|) |  |
| --- | --- | --- | --- | --- | --- | --- |
| (Intercept) | β0 | 0.3285627 | 0.0480316 | 6.841 | 2.11x10^-6^ | *** |
| Future percentage of seizure reduction (after one year of VNS) | β1 | -0.0006967 | 0.0003720 | -1.873 | 0.0774 | . |
| Patient’s age at EEG | β2 | 0.0022656 | 0.0008004 | 2.830 | 0.0111 | * |
| Epilepsy duration | β3 | -0.0004939 | 0.0007568 | -0.653 | 0.5223 |  |
| *Sex* | *β4* | *0.0124181* | *0.0205232* | *0.605* | *0.5527* | *Higher p-value* |
| Number of ASM | β5 | 0.0171700 | 0.0128470 | 1.336 | 0.1980 |  |
| Type of epilepsy | β6 | 0.0157488 | 0.0199630 | 0.789 | 0.4404 |  |

*: p<0.05　　**: p<0.01　　***: p<0.001

Abbreviations: VNS (vagus nerve stimulation), EEG (electroencephalogram), ASM (antiseizure medication), BZD (benzodiazepine)

Model 4: 5 variables

Adjusted R-squared of the model: 0.2982

p-value: 0.03507*

| Explanatory variable | Para-  meter | Estimate | Standard error | t value | Pr (>\|t\|) |  |
| --- | --- | --- | --- | --- | --- | --- |
| (Intercept) | β0 | 0.3326953 | 0.0467438 | 7.117 | 9.09x10^-7^ | *** |
| Future percentage of seizure reduction (after one year of VNS) | β1 | -0.0007517 | 0.0003546 | -2.120 | 0.04743 | * |
| Patient’s age at EEG | β2 | 0.0023243 | 0.0007812 | 2.975 | 0.00777 | ** |
| *Epilepsy duration* | *β3* | *-0.0004816* | *0.0007438* | *-0.647* | *0.52511* | *Higher p-value* |
| Number of ASM | β4 | 0.0171700 | 0.0126164 | 1.390 | 0.18047 |  |
| Epilepsy type | β5 | 0.0163519 | 0.0196027 | 0.834 | 0.41455 |  |

*: p<0.05　　**: p<0.01　　***: p<0.001

Abbreviations: VNS (vagus nerve stimulation), EEG (electroencephalogram), ASM (antiseizure medication), BZD (benzodiazepine)

Model 5: 4 variables

Adjusted R-squared of the model: 0.3248

p-value: 0.01438*

| Explanatory variable | Para-  meter | Estimate | Standard error | t value | Pr (>\|t\|) |  |
| --- | --- | --- | --- | --- | --- | --- |
| (Intercept) | β0 | 0.3326953 | 0.0447856 | 7.414 | 2.73x10^-7^ | *** |
| Future percentage of seizure reduction (after one year of VNS) | β1 | -0.0008114 | 0.0003250 | -2.497 | 0.02092 | * |
| Patient’s age at EEG | β2 | 0.0020996 | 0.0006688 | 3.139 | 0.00495 | ** |
| Number of ASM | β3 | 0.0182658 | 0.0114742 | 1.592 | 0.12635 |  |
| *Epilepsy type* | *β4* | *0.0123343* | *0.0177529* | *0.695* | *0.49481* | *Higher p-value* |

*: p<0.05　　**: p<0.01　　***: p<0.001

Abbreviations: VNS (vagus nerve stimulation), EEG (electroencephalogram), ASM (antiseizure medication), BZD (benzodiazepine)

Model 6: 3 variables

Adjusted R-squared of the model: 0.3407

| Explanatory variable | Para-  meter | Estimate | Standard error | t value | Pr (>\|t\|) |  |
| --- | --- | --- | --- | --- | --- | --- |
| (Intercept) | β0 | 0.3391701 | 0.0430800 | 7.873 | 7.69x10^-8^ | *** |
| Future percentage of seizure reduction (after one year of VNS) | β1 | -0.0008481 | 0.0003168 | -2.677 | 0.01378 | * |
| Patient’s age at EEG | β2 | 0.0020808 | 0.0006604 | 3.151 | 0.00464 | ** |
| Number of ASM | β3 | 0.0175938 | 0.0112981 | 1.557 | 0.13369 |  |

p-value: 0.006631**

*: p<0.05　　**: p<0.01　　***: p<0.001

Abbreviations: VNS (vagus nerve stimulation), EEG (electroencephalogram), ASM (antiseizure medication), BZD (benzodiazepine)
