# Supplementary material for: Electroencephalogram synchronization measure as a predictive biomarker of Vagus nerve stimulation response in refractory epilepsy: A retrospective study
Source: PLoS One. 2024 Jun 11;19(6):e0304115. doi: 10.1371/journal.pone.0304115 (PMC11166337; doi:10.1371/journal.pone.0304115)
Supplement: Attachment — Submitted filename: Response to Reviewers.pdf [file pone.0304115.s004.pdf]

Manuscript PLOS ONE [PONE-D-23-43902]: "Electroencephalogram Synchronization Measure as a Predictive Biomarker of Vagus Nerve Stimulation Response in Refractory Epilepsy: A Retrospective Study"

Authors response

Dear Editor, dear Reviewers,

Thank you very much for your valuable feedback and comments on our submitted manuscript.

In this document, we have addressed all comments by providing a response and, if necessary, the corresponding changes in the related text and figures. Changes in the original text are underlined in yellow.

Here is a point-by-point response to your comments.

Reviewer Comments: Reviewer #1

Comments to the Author

**1**	**General Comment**	The study involved an analysis of 38 patients who underwent VNS implantation, aiming to identify predictive factors for VNS response by comparing EEG data between responders and non-responders. In particular, the authors assess brain synchronization by wPLI, considering that desynchronization is one of the mechanisms of VNS. Notably, the study uncovered an association between low wPLI during sleep and favorable seizure reduction. Given the absence of predictive biomarkers for VNS, this discovery holds substantial implications for clinical practice. However, the Introduction and Discussion are somewhat redundant, making it difficult to understand the main message of this study. Additionally, the presentation of results lacks adequate detail. Significant revisions are necessary before publication.
	Response	We appreciate your feedback regarding our work, and the interesting aspects pointed out in order to improve the manuscript quality. Those comments are of great assistance to us for improving and revising our manuscript.
**2**	**Comment**	[Introduction/Discussion] Please describe the strengths or novelty of this research. What is the strong point compared to the previous studies with synchronization measurements such as PLI or wPLI?

	Response	We acknowledge the reviewer's observation that the initial text could be improved to better highlight the strengths of this study. Therefore, the text has been adapted accordingly (see changes in section 'related text'). In addition to the details in the text, the strengths and novelty of the study are described hereunder.<br><br>Previous research has primarily focused on PLI and wPLI following VNS implantation (Sangare et al. 2020, Bodin et al. 2015, Vespa et al. 2021). Broadly, these studies indicate a greater desynchronization in responders (R) compared to non-responders (NR). In addition, as highlighted in the introduction (lines 82-86), this desynchronization has been also observed to be specifically significant in sleep (Vespa et al 2021).<br><br>This interest in sleep stems from the underlying physiology of sleep, particularly stage 2 sleep (the focus sleep stage on our study as well). Indeed, during NREM sleep the brain circuits entrain thalamo-cortical oscillatory phenomena (such as sleep spindles and, to a lesser extent, K-complexes). These oscillations have been theorized to mirror certain thalamocortical circuits implicated in the generation of pathological epilepsy oscillatory phenomena, such as spike-wave discharges. We refer to specific reviews, Sinha et al. (2011) and Bernard et al. (2023), for explanations about the physiological hypotheses justifying this correlation. Moreover, there exists a substantial body of evidence supporting the concept that the modulation of thalamo-cortical connections could be a key mechanism of acute VNS effect, as demonstrated in studies by Vonck et al. (2008), Ibrahim et al. (2017), and Hachem et al. (2018).<br><br>However, despite these significant findings, the number of studies investigating wPLI or PLI before VNS implantation remains limited (Ma et al. 2022 and Fraschini et al. 2013) and the results remain inconclusive. Thus, our study not only contributes to adding a novel investigation of wPLI in pre-implantation DRE patients but also constitutes investigations of wPLI *during sleep* in these patients. To our knowledge, no previous study investigated wPLI during sleep in patients with DRE before VNS implantation. Given the physiological peculiarities associated with sleep, this could potentially lead to the identification of a specific marker for predicting the response to VNS. In response to your comment, we

		believe that incorporating a sentence to describe the novelty of the work will indeed enhance the overall quality of our work. We have integrated these aspects into our revisions accordingly.
	Related text	**Section 1: Introduction**<br><br>More details about sleep have been added in the original text to emphasize the novelty of our research investigating wPLI in DRE patients during sleep (lines 62-67 and lines 95-98).<br><br>Lines 63-68: "In particular, one study has specifically noted that this desynchronization occurs during sleep (Vespa et al. 2021). This interest in sleep stems from sleep physiology, particularly stage 2 of sleep. Indeed, during NREM sleep, thalamo-cortical oscillatory phenomena – including sleep spindles and, to a lesser extent, K-complexes - are observed, and are typically found during sleep stage N2 (Steriade et al. 1993, Platt et al. 2011, Purcell et al. 2017). These oscillations have been theorized to mirror certain thalamocortical circuits implicated in the generation of pathological epilepsy oscillatory phenomena, such as spike-wave discharges (Sinha et al. 2011 and Bernard et al. 2023). "<br><br>Lines 94-99: "Consequently, in order to develop predictive biomarkers of VNS response, this study aims to extract EEG-based synchronization metrics before VNS implantation and compare them during sleep and wakefulness, between R and NR. This study constitutes the first investigation using the wPLI – a metric known to be more sensitive to true neural synchronization due to its insensitivity to noise (Congedo et al. 2018, Vinck et al. 2011) - to investigate connectivity in DRE patients during wakefulness and sleep before the implantation of a VNS device."
**3**	**Comment**	[Introduction] The explanation of the mechanism of VNS seems redundant. There is no need to use several paragraphs to mention it.
	Response	Thank you for bringing this aspect of the manuscript to our attention.<br><br>We adapted the text to only keep important details for understanding the results presented as part of our study and removed any redundancy.

<table>
<tr><td></td><td>Related text</td><td>We adapted the text according to the comment of the reviewer. Redundancy has been removed. Mechanisms of action have been summarized in lines 47-51.

Section 1: Introduction

<u>Revised (modified) text:</u>
Lines 48-52: “Using various recording techniques - including low-density scalp EEG (Bodin et al. 2015; Sangare et al. 2020; Vespa et al. 2021) or stereo-EEG (Bartolomei et al. 2016), it has been suggested that VNS reduces seizure susceptibility by decreasing excitability of the cortex. Indeed, VNS is thought to partially exert its anti-seizure effects through the activation of widespread thalamocortical connections via the activation of the Locus Coeruleus (LC) - noradrenergic system (Berger et al. 2021), receiving projections from the Nucleus Tractus Solitarius (NTS) (Krahl et al. 2012).”</td></tr>
<tr><td>4</td><td>Comment</td><td>[Introduction] It’s undesirable to use the term “hypothesize” to describe the ideas of other authors or articles. It would be better to use “hypothesize” only when describing your research hypothesis.</td></tr>
<tr><td></td><td>Response</td><td>The word “hypothesize” has been removed in this context.</td></tr>
<tr><td></td><td>Related text</td><td><u>Revised (modified) text:</u>

- Line 48-49: “Using various recording techniques - including low-density scalp EEG (Bodin et al. 2015; Sangare et al. 2020; Vespa et al. 2021) or stereo-EEG (Bartolomei et al. 2016), it has been suggested that VNS reduces epileptic susceptibility by decreasing excitability of the cortex.”

- Line 81-82: “The authors suggested that differential neuronal excitability and synaptic transmission could exist among patients, and could influence the therapeutic response (Brázdil et al. 2019).”

- Lines 297-299: “The authors suggested that an increased global efficiency could reflect a more extensive</td></tr>
</table>

<table>
<tr><td></td><td></td><td>pathological (epileptic) network within the brain (Carboni et al. 2020)."

- Lines 308-309: "Hence, the authors suggested that an over-connectivity may reflect a more pathological network (Davis et al. 2019)."</td></tr>
<tr><td>5</td><td>Comment</td><td>[Introduction] Please add more explanation of wPLI or PLI. Could you explain why these measurements are useful, citing past reports? It would clarify the importance of using these measurements in this study.</td></tr>
<tr><td></td><td>Response</td><td>Thank you for your comment. You will find hereunder more detailed information about the different connectivity measures that have been used in previous studies. Moreover, we emphasized in the text the choice of wPLI compared to other connectivity measures (see 'related text').

The current study employed connectivity measures to investigate the functional brain interconnectivity in an attempt to predict VNS response prior to implantation. Among these measures, one particular metric (wPLI) stood out due to its ability to assess desynchronization following VNS implementation. Particularly, metrics focusing on phase synchronization difference have been frequently utilized to characterize the impact of VNS on the brains of DRE patients, after VNS implantation, revealing desynchronization in VNS responders (R) compared to non-responders (NR), as observed in studies by Vespa, Bodin or Sangare. Whereas these post-implantation studies were referenced in the discussion, the introduction primarily emphasized pre-implantation investigations, as highlighted by Ma et al. and Fraschini et al.

While previous studies at first utilized PLV (phase locking value) or PLI (phase locking index), it has been demonstrated that another synchronization metric, known as wPLI (weighted phase lag index), could be advantageous. Indeed, this metric is less susceptible to noise between electrodes and may therefore offer greater sensitivity to true neural synchronization, as suggested by</td></tr>
</table>

		Vinck and Congedo (Vinck et al. 2011, Congedo et al. 2018). The mathematical definition of the wPLI can be found in lines 149-160.
	Related text	**Section 1: Introduction**<br><br>More information about the use of these metrics in previous reports and details about the importance of using them were added to the manuscript.<br><br>**<u>Revised (modified and added) text:</u>**<br><br>Lines 59-62: "While various FC measures exist, the metrics focusing on the coupling of signal phases are known as phase synchronization measures (Van Mierlo et al. 2014). Previous studies that used EEG-based phase synchronization measures (weighted Phase Lag Index – wPLI, Phase Lag Index – PLI, or Phase Locking Value - PLV) revealed a desynchronization effect with acute VNS administration (Bodin et al. 2015; Sangare et al. 2020; Vespa et al. 2021)."<br><br>Lines 96-99: "This study constitutes the first investigations using the wPLI – a metric known to be more sensitive to true neural synchronization due to its insensitivity to noise (Congedo 2018; Vinck et al. 2011) - to investigate connectivity in DRE patients during wakefulness and sleep before the implantation of a VNS device."
**6**	**Comment**	[Results] Due to the lack of explanation, it is hard to understand the results. An example is "For the delta band, the LMM revealed a significant effect of state (wakefulness/ sleep) (p=0.016*) but no effect of VNS response (p=0.29) nor an interaction between state and VNS response (p=0.27)."<br><br>・Does "a significant effect of state (wakefulness/sleep)" mean that there is a significant difference in wPLI between wake and sleep? Which states have higher wPLI?<br><br>・Does "no effect of VNS response (p=0.29)" mean that there is no difference in wPLI between VNS responders and non-responders?

		・What does "an interaction between state and VNS response (p=0.27)" mean specifically?<br>Please describe the results in more detail or use Table, not just in this example but all results.
	Response	The results section has been revised for clarity. We have re explained the main findings of our study below and a thorough discussion of the results can be found in the discussion section. Moreover, as suggested by the reviewer, Tables summarizing the results of the LMM models have been created and added in the Supplementary Materials (S1 – results for the delta band, and S2 – for the alpha band).<br><br>First, a summary of the main findings:<br><br>- In the delta band, a higher wPLI during wakefulness compared to sleep was observed in NR only.<br>- The only band capable of discriminating R and NR is alpha band in sleep with a trend of higher wPLI in NR compared to R.<br>- A multiple linear regression analysis conducted in patients for whom the exact reduction in seizure frequency is known, revealed that a lower pre-implantation wPLI during sleep in alpha band was associated with a better response to VNS.<br><br>More details about the analyses can be found hereunder.<br><br>We conducted a Linear Mixed Model (LMM) analysis to investigate how the state (wakefulness/sleep) and response (R/NR) affects wPLI (weighted phase lag index) in patients, separately for each frequency band.<br><br>The Linear Mixed Models with interaction were formulated as follows: $wPLI_{band}$ ~ State + Response + State*Response + (1|id)<br><br>With:<br>- "band" referring to the different frequency bands (delta, theta, alpha, beta, broadband)<br>- "State" referring to sleep and wakefulness

- “Response” referring to R and NR.
- "id" represents the random effect for individual patients, which helps account for inter-subject variability.

This equation allowed us to evaluate the relationship between state and wPLI values, as well as response and wPLI values. In this model, the state and the response are incorporated as independent variables, and the wPLI as the dependent variable.
We adjusted for the impact of covariates in the models; however, no significant outcomes were observed (refer to S1/S2 in the supplementary material). The parameter estimates for each fixed effect in the model are shown in Supplementary Materials S1 for the delta band and S2 for the alpha band.
Utilizing LMM allows us to conduct post-hoc tests, known as contrasts, within the model. These contrasts enable specific comparisons of interest between levels of categorical variables in the LMM and built for each frequency band of interest.

Nevertheless, our binary classification approach (R/NR with a threshold at 50% of seizure reduction) may introduce potential biases due to the exact percentage of seizure reduction not being considered. For instance, patients with a 49% seizure reduction are classified as NR, yet they may still exhibit a certain level of response to VNS. This could potentially influence the results. This discrepancy may explain why the interaction in the LMM of alpha band was not significant, and only a trend was observed in the contrast test between R and NR. However, when considering the exact percentage of seizure reduction, differences between R and NR could become apparent. To address this issue, we calculated the actual percentage of seizure reduction in patients whenever it was possible (27 out of 38 patients).

The multiple linear regression was computed using the following equation (Statistical comparison of the linear regressions can be found in Supplementary material S3): $wPLI_{alpha/sleep}$~ Percentage of seizure reduction + age + Number of ASM

With:

- Alpha/sleep referring to wPLI in alpha band in sleep only.

		- Percentage of seizure reduction referring to the exact percentage of seizure reduction instead of a binary response.<br>- Age referring to the age of the patient at the time of the EEG.<br>- Number of ASM referring to the number of Anti-seizure Medication (ASM) taking by the patient at the moment of the EEG.<br><br>This equation allowed us to evaluate the relationship between wPLI value in alpha band in a specific state (here sleep since the trend in LMM was found only in alpha band in sleep) and some covariates (including age and number of ASM), as well as the exact percentage of seizure reduction.<br><br>We adjusted for the impact of covariates in the models; however, no significant outcomes were observed for the other covariates (refer to S3 in the supplementary material).
	Related text	**Section 3.2.1: (Results) Whole brain analysis**<br><br>**<u>Revised (modified and added) text:</u>**<br><br>Lines 225-235: "In the delta band analysis, the LMM revealed that the state variable exhibited a significant effect on wPLI **(p=0.016*)**, with lower wPLI values found in sleep compared to wakefulness (Supplementary Material S1 Table). On the other hand, the response variable showed no significant impact on wPLI (p=0.29), indicating no difference in wPLI between responders (R) and non-responders (NR). Moreover, the interaction between State and Response was not significant (p=0.27), suggesting that our model cannot distinguish between R and NR within each state in the delta band. Utilizing LMM allows us to conduct post-hoc tests, known as contrasts, within the model. These contrasts enable specific comparisons of interest between levels of categorical variables in the LMM. In the delta band model, contrasts revealed that higher wPLI values were observed during wakefulness compared to sleep, but only for non-responders **(p=0.022*)**. No significant difference in wPLI between wakefulness and sleep for responders was found (p=0.71). Please refer to Fig 2 for a visual representation."

		Lines 239-244:" In the analysis of the alpha band, a significant effect of the state on wPLI **(p= 0.044*)** is found, while neither the response (p=0.57) nor the interaction showed significant impacts (p= 0.17). We did not observe any significant results in the contrast analysis. However, a trend emerged during sleep between responders (R) and non-responders (NR) (p = 0.058), with NR exhibiting a higher wPLI compared to R, while no trend is found in wakefulness (p=0.57). Please refer to Fig 3 for a visual representation."<br><br>Lines 248: "More detailed information about the LMM can be found in Supplementary Materials S1 and S2 tables."<br><br>Lines 251-263: "To mitigate biases inherent of a binary classification and investigate in more detail the trend found in alpha band in sleep, our aim was to address the specific percentage of seizure reduction through a regression analysis.<br>For patients in whom the exact percentage of seizure reduction was available (N=27), the multiple linear regression in the alpha band during sleep revealed a significant association between percentage of seizure reduction **(p = 0.014*)** and global wPLI, indicating a decrease in wPLI among patients exhibiting a stronger response to VNS (i.e., greater seizure reduction). Secondly, age demonstrated a significant correlation with wPLI **(p=0.005**)**, revealing higher wPLI levels among older patients. Interestingly, the number of antiseizure medications (ASMs) did not yield a significant effect on wPLI but contributed to enhancing the accuracy of the model (adjusted R-squared: 0.34; statistical comparison of the linear regressions can be found in Supplementary Material S3). For visual representation, a linear regression between the wPLI in the alpha band during sleep and the percentage of seizure reduction is shown in Fig 4."
**7**	**Comment**	[Results] It would be better to add the information that a high wPLI means increased connectivity in addition to the results (Is my interpretation correct?). This is because many readers would be expected not to know what high wPLI means.
	Response	Thank you for this comment. The reviewer's interpretation is indeed accurate and has been addressed in the manuscript.

		More details about wPLI have been added in the Materials and Methods section (see ‘related text’).<br><br>Moreover, interpretation of a higher wPLI has been addressed in the discussion section (i.e. higher wPLI may suggest increased connectivity).
	Related text	**Section 2.2: (Materials and Methods) Connectivity Analysis: EEG synchronization measures**<br><br>**<u>Revised (added and modified) text:</u>**<br><br>Lines 143-149: "As we introduced earlier, amongst the various existing connectivity measurements, we have chosen to focus on the phase-synchronization measures. These measures are used to quantify phase synchronization between EEG signals (Hu and Zhang n.d.; Lachaux et al. 1999). The PLI is one of the most well-known metrics. This metric estimates the asymmetry of distribution of phase differences between two signals and is partially corrected for volume conduction (Stam et al. 2007). The wPLI improves the PLI by weighting the phase differences by their magnitude, reducing the risk of bias introduced by small noise perturbations and making the wPLI more sensitive to detect true neural synchronization (Congedo 2018; Vinck et al. 2011).<br><br>**Section 4: Discussion**<br><br>**<u>Revised (added) text:</u>**<br><br>Line 272-277:” Using wPLI as a connectivity metrics (as wPLI is an accepted marker of connectivity (Van Mierlo et al. 2014), this study attempts to identify predictive markers of VNS response by analyzing pre-implantation EEG data during wakefulness and sleep. Despite the absence of a clear predictive marker for VNS efficacy, this study points to certain physiological factors that appear to impact the therapeutic response. Indeed, we found a higher wPLI (i.e. may be interpreted as higher FC) in the delta band during wakefulness compared to sleep, especially in NR.”

**8**	**Comment**	[Figure 2-4 legend] It would be nicer to describe an explanation of each result in Figure legend.
	Response	Thank you for highlighting this point. The legends of the Figures have been revised.
	Related text	**Section 3.2.1: (Results) Connectivity Analysis: EEG synchronization measures – Whole brain analysis**<br><br>**<u>Revised (added and modified) text:</u>**<br><br>Lines 179-180: ***"Fig 1**: **Regional electrode pooling for connectivity analysis**. Seven different regions were defined: three per hemisphere (frontal, parietal and occipital) and one central region* (Vespa et al. 2021).*"*<br><br>Lines 236-238: ***"Fig 2: Boxplots of the wPLI in the delta band. (A)** NR patients demonstrated a higher wPLI in wakefulness compared to sleep while **(B)** no difference is found between states in R patients. ★ Significant result at the level p<0.05 in post-hoc comparison."*<br><br>Lines 245-247: ***"Fig 3**: **Boxplots of the wPLI in the alpha band. (A)** In wakefulness, no difference was found between groups while **(B)** in sleep, a higher wPLI is found in NR group compared to R. ✫ Trend of significance in post-hoc comparison (p= 0.058)."*<br><br>Lines 264-266: ***"Fig 4: Linear regression between wPLI in the alpha band during sleep and the percentage of seizure reduction**. During sleep, a lower wPLI was observed in patients with a higher reduction in seizure frequency. "*
9	Comment	[Figure 3] It is not common to use symbols such as asterisks for results that are not significantly different. Please remove it as it is misleading.
	Response	Thanks for this precision. We have changed the symbols to avoid confusion.
	Related text	**Section 3.2.1: (Results) Connectivity Analysis: EEG synchronization measures – Whole brain analysis**<br><br>**<u>Revised (added) text:</u>**

		Lines 236-238: ***"Fig 2: Boxplots of the wPLI in the delta band.*** *[...]. ★ Significant result at the level p<0.05 in post-hoc comparison."*<br>Lines 245-247: *"**Fig 3**: **Boxplots of the wPLI in the alpha band.** [...] ☆ Trend of significance in post-hoc comparison (p= 0.058)."*<br><br>Figure 2 and Figure 3 have also been adapted accordingly.
10	Comment	[Discussion] Given that brain connectivity increases during sleep, wPLI during wake is considered to be lower than during sleep. Please discuss why the phenomenon opposite to the theory occurred.
	Response	More information about these findings is described here. Moreover, more details have been added to the text (see changes in 'related text').<br><br>We acknowledge that this point may initially seem counter-intuitive, as noted by the reviewer. However, it is crucial to recognize that this phenomenon varies across frequency bands. In healthy individuals, wPLI typically increases in the delta band during sleep compared to wakefulness, whereas it decreases in the alpha band during sleep. Our findings in patients exhibit a contrasting pattern, with: 1) higher wPLI in wakefulness compared to sleep in the delta band for NR, and 2) in alpha band, in sleep, a higher wPLI is found in NR compared to R. Additionally, certain pre-implantation studies suggest that R may exhibit connectivity profiles more similar to healthy subjects than NR. For example, as discussed, Kim et al. observed a similar profile between R and healthy subjects, whereas NR showed greater divergence from the healthy population. Similarly, two MEG studies highlighted a closer connectivity pattern between R and controls compared to NR (Mithani et al. 2019, Hachem et al. 2018).<br><br>Overall, both these studies and our findings converge on a similar conclusion: the occurrence of a phenomenon opposite to the expected theory is observed in NR, which aligns with existing literature.<br><br>Furthermore, given that R patients also exhibit characteristics of DRE patients, it is plausible that their brain circuitry might be

		pathological, albeit to a lesser degree than NR patients. Therefore, it is understandable that R patients do not demonstrate the same connectivity patterns as described above in healthy individuals.<br><br>To highlight the distinction between the frequency bands, we revised the abstract to underscore that the heightened synchronicity during sleep specifically occurred in the delta band. Conversely, alpha band synchronicity increases during wakefulness. This clarification is further elucidated in the discussion section.
	Related text	**Section: Abstract**<br><br>**<u>Revised (added and modified) text:</u>**<br><br>Lines 19-26: "There are currently no established biomarkers for predicting the therapeutic effectiveness of Vagus Nerve Stimulation (VNS). Given that neural desynchronization is a pivotal mechanism underlying VNS action, EEG synchronization measures could potentially serve as predictive biomarkers of VNS response. Notably, an increased brain synchronization in delta band has been observed during sleep – potentially due to an activation of thalamocortical circuitry, and interictal epileptiform discharges are more frequently observed during sleep. Therefore, investigation of EEG synchronization metrics during sleep could provide valuable insight into the excitatory-inhibitory balance in a pro-epileptogenic state, that could be pathological in patients exhibiting a poor response to VNS."<br><br>**Section 4: Discussion**<br><br>**<u>Revised (added and modified) text:</u>**<br>Lines 287-292: "Interestingly, no differences between wakefulness and sleep was found amongst patients in global wPLI for the alpha band activity. These findings were somewhat unexpected, considering the existing literature that underscores the significance of alpha rhythm in consciousness in healthy patients. At first glance, our results seem to be inconsistent with previous findings in healthy participants in whom a higher connectivity (as assessed with wPLI - within the alpha band was observed during

		wakefulness compared to sleep. In contrast, the enhancement of wPLI during sleep was found in the delta band instead (48–50)"
11	Comment	[Discussion] The idea that more pathological connectivity is related to VNS non-responders seems to be inconsistent with the VNS mechanism. This is because VNS appears to alleviate the symptoms of epilepsy patients with high connectivity (=more pathological connectivity) through desynchronization. Please give a convincing explanation.
	Response	Thank you to the reviewer for highlighting this intriguing observation. Indeed, one of our initial hypotheses could have aligned with the suggestion made by the reviewer. However, an alternative explanation could be that if NRs exhibit an inverse synchronization pattern (higher alpha band activity during sleep and delta band activity during wakefulness), it might indicate more severe pathology in their thalamo-cortical (TC) circuits, as stage 2 sleep reflects their functioning. Notably, VNS targets these circuits, suggesting that in cases of excessively pathological TC circuits, VNS efficacy might be compromised. This could elucidate the lower weighted phase lag index (wPLI) observed in R patients, as VNS might exert greater influence due to less pathological circuitry.<br><br>Additionally, previous studies reporting desynchronization primarily focused on post-implantation patients, comparing acute desynchronization (ON vs. OFF VNS) in R vs. NR patients. To the best of our knowledge, no study has compared pre- and post-implantation patients using phase synchronization measures similar to ours. Consequently, the baseline synchrony levels before VNS implantation remain unknown. It is plausible that both R and NR populations exhibit higher synchrony before implantation, with R patients experiencing a more pronounced decrease following VNS administration compared to NR patients.<br><br>Importantly, this interpretation does not contradict our findings, which underscore a pre-existing difference prior to VNS implantation, without assessing the acute effect of VNS on brain synchronization. Therefore, we believe our results are not inconsistent with post-implantation studies evaluating VNS-desynchronizing effects.

		We hope that these explanations provide clarity for the reviewer. We have adjusted the discussion section to emphasize these clarifications accordingly.
	Related text	This interesting point has been addressed when discussing the future prospects of our study:<br><br>Lines 364-367: "Given [...] and the interest of following patient before and after the VNS surgery, we propose a longitudinal follow-up study to address the temporal variability of the wPLI and the impact of VNS on this measure."
12	Comment	[General comment] Please double-check the grammar and logical development of the manuscript again.
	Response	As suggested by the reviewer, the manuscript has been checked for grammar and clarity to improve readability.
13	Comment	・Brackets of "(Video-)EEG recordings were…" in 109 seems to be unnecessary.<br>・The use of the term "future" VNS response feels inappropriate in a retrospective study.<br>・Table 1: The use of "n°" is not common. It would be more understandable to use "no." or "n"(italic).<br>・Table 1: It would be better to change "<2.2e-16" to "2.2×10-16" or "<0.001".<br>・Results: What does the asterisk (for example, p=0.006*) mean? If unnecessary, please remove it.
	Response	Thanks for pointing out these details. Each point has been addressed hereunder.<br><br>• Brackets are present because some patients did not have the video but only EEG recording in the case of patients who were not candidate to surgery.<br><br>• We thank the reviewer of pointing this out and have deleted the term "future" VNS responders (See 'related text').<br><br>• Table 1 has been adapted according to the comment of the reviewer (See 'related text').<br><br>• Thank you for pointing this, correction has been made (See 'related text').

	Related text	(see below)

Related text

- The term “future” N/NR has been removed in the text and in corresponding figures to avoid confusion.

- **Table 1. Demographic and clinical characteristics of the study population.**

Variables	***R (n= 12) Mean (SD)***	***NR (n=26) Mean (SD)***	***p-value***
*Sex**	7F – 5M	13F – 13M	0.92
Age (years)	37.9 (12.8)	37.4 (13.6)	0.92
Age of Epilepsy onset (years)	16 (11.07)	14.24 (16.5)	0.71
Epilepsy duration (years)	21.18 (16.2)	23.52 (16.5)	0.70
Epilepsy type			<2.2×10-16
Focal	12	18	
Generalized	0	7	
Both	0	1	
Localization			0.66
Temporal (T)	7	7	
Extra-temporal (ET)	4	16	
Both	1	3	
Mean no. of ASMs used	3 (1.17)	2.6 (0.77)	0.24
No. using benzodiazepines	3/12	3/26	0.38
No. using antidepressants (SSRI, TCA)	3/12	4/26	0.38
Epilepsy lateralization			0.75
Right	1	4	
Left	6	12	
Bilateral	5	10	

Legend: F: Females; M: Males; ASM: Anti-Seizure Medication; SSRI: Selective Serotonin Reuptake Inhibitor; TCA: Tricyclic Antidepressant Agent

		**Sex assigned at birth*<br><br>• The usefulness of a random variable in the model has been confirmed in two bands: delta **(p=0.006**)** and alpha **(p=0.04*)** bands.

Reviewer Comments: Reviewer #2

Comments to the Author

**1**	**General Comment**	Danthine et al. studied a predictor for clinical response to VNS in epileptic patients using EEG phase synchronization metrics. They mentioned DRE patients, in particular future non-responders, have a greater functional connectivity in the delta band during wakefulness compared to sleep.<br><br>We want to congratulate the authors' efforts. The manuscript is well written and easy to follow. None of the following comments are criticisms.
	Response	We thank you for your comments.
**2**	**Comment**	Abstract (line 28): Wouldn't the statement "especially in NR" be unnecessary since you later say that the difference between R and NR did not reach significance?
	Response	Thanks for pointing out this point. The abstract has been modified for more clarity.<br><br>Indeed, while no difference between R and NR was found, a higher wPLI in wakefulness compared to sleep was found in NR only.
	Related text	**Section: Abstract**<br><br>**Revised (modified) text:**<br>Lines 31-32: "In the delta band, a higher wPLI was found during wakefulness compared to sleep in NR only. However, in this band, no synchronization difference was found in any state was found between R and NR."
**3**	**Comment**	Introduction (line 86): It would be preferable to spell out the abbreviations for PSG when it is first mentioned.
	Response	Thank you for spotting this issue, the abbreviation has been added in the text.
	Related text	**Section 1: Introduction**

		**<u>Revised (added) text:</u>**<br><br>Lines 91-93: "Indeed, polysomnography (PSG) findings suggest that sleep quality correlates with a less severe epilepsy and better clinical effects in DRE (Proost et al. 2022; Zanzmera et al. 2012)."
4	Comment	Introduction (line 90-91): I understand what the authors are saying. And indeed, as the authors report, there was a negative correlation between the wPLI and the future percentage of seizure reduction after VNS implantation, only during sleep. In that case, it is natural to assume that changes have occurred in the postoperative EEG. Have you measured the postoperative EEG? If you did, please discuss this point.
	Response	We recognize the value in investigating post-implant sleep EEG to assess whether VNS can potentially reverse the effects observed in our study. However, it is important to note that the majority of patients, particularly R patients, do not undergo new video-EEG monitoring post-implantation. Consequently, the available EEG data for analysis would be biased, as only NR patients would typically have such data accessible. Additionally, anti-seizure medications (ASM) are often not consistently maintained, which could introduce further bias into the results.<br><br>Due to these limitations, we discussed about the future prospects of our research in the discussion section. Indeed, a longitudinal study design was proposed, where patients are followed before and after VNS implantation, striving to maintain ASM as stable as possible. We have adjusted the discussion section to appropriately address this proposed approach.
	Related text	**Section 4: Discussion**<br><br>**<u>Revised (added) text:</u>**<br>Lines 364-367: "Given the inherent patient-to-patient variability and the interest of following patient before and after the VNS surgery, we propose a longitudinal follow-up study to address the temporal variability of the wPLI and the impact of VNS on this measure."
5	Comment	Figure1 (line 168-169): The author may want to write "T4-C4" instead of "T4,Ca".
	Response	Thank you for recognizing this typo.

	Related text	*Corrected Fig1.:*<br>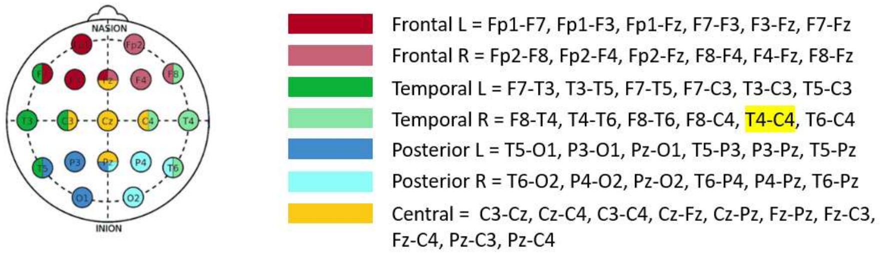
6	Comment	Results (line 203 -204): R group is only focal epilepsy. I would appreciate it if you could discuss whether this affected the main finding (the difference in synchronization between two groups).
	Response	We appreciate the reviewer for bringing up this question. Indeed, this point had indeed been raised during realization of this study. Firstly, it is noteworthy that most studies include both focal and generalized epilepsies within each group (R and NR) (Bodin et al. 2015, Vespa et al. 2021).<br><br>The imbalance between focal and generalized epilepsy patients in the NR and R groups may be attributed to the small sample size. However, Sangare investigated the PLI difference between focal and generalized epilepsy and no difference was found (Sangare et al. 2020). Lastly, to ensure thorough consideration of this factor, we incorporated it as a variable in our statistical models to remove possible effects of epilepsy type on wPLI investigations. Notably, this covariate exhibited no significance in either our mixed model (Supplementary Materials S1 and S2) or our regression model (Supplementary Material S3).<br><br>We have incorporated a paragraph into the discussion section to point out this aspect.
	Related text	**Section 4: Discussion**<br><br>**Revised (added) text:**<br>Lines 354-361: "Additionally, in our dataset, a significant distinction arises between responders (R) and non-responders (NR) concerning epilepsy type. Nevertheless, we maintain that this factor does not impact our primary findings. Indeed, this imbalance of epilepsy type between the groups could be due to our relatively low sample size, while previous studies did not find association between VNS response and epilepsy type (Bodin et al. 2015, Vespa et al. 2021). Futhermore, Sangare et al. investigated the PLI difference between focal and

		generalized epilepsy and no difference was found (Sangare et al. 2020). Moreover, we have addressed this variable in our models to ensure its control in our statistical analyses. No effect of epilepsy type was found in the mixed model analysis or in the regression model analysis (Supplementary Material S3)."
